# Autophagy-associated immune responses and cancer immunotherapy

**DOI:** 10.18632/oncotarget.6908

**Published:** 2016-01-13

**Authors:** Hongming Pan, Liuxi Chen, Yinghua Xu, Weidong Han, Fang Lou, Weiqiang Fei, Shuiping Liu, Zhao Jing, Xinbing Sui

**Affiliations:** ^1^ Department of Medical Oncology, Sir Run Run Shaw Hospital, Zhejiang University, Hangzhou, China; ^2^ Biomedical Research Center and Key Laboratory of Biotherapy of Zhejiang Province, Hangzhou, China

**Keywords:** autophagy, immune, cancer immunotherapy

## Abstract

Autophagy is an evolutionarily conserved catabolic process by which cellular components are sequestered into a double-membrane vesicle and delivered to the lysosome for terminal degradation and recycling. Accumulating evidence suggests that autophagy plays a critical role in cell survival, senescence and homeostasis, and its dysregulation is associated with a variety of diseases including cancer, cardiovascular disease, neurodegeneration. Recent studies show that autophagy is also an important regulator of cell immune response. However, the mechanism by which autophagy regulates tumor immune responses remains elusive. In this review, we will describe the role of autophagy in immune regulation and summarize the possible molecular mechanisms that are currently well documented in the ability of autophagy to control cell immune response. In addition, the scientific and clinical hurdles regarding the potential role of autophagy in cancer immunotherapy will be discussed.

## INTRODUCTION

**Figure 1:**
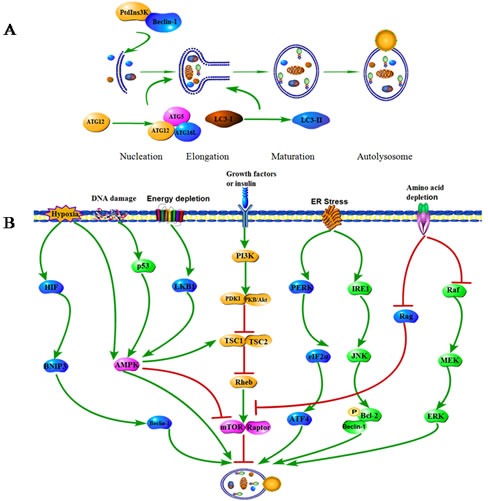
Phases and molecular regulation of autophagy **A.** The four main phases of autophagy. Autophagy proceeds through several phases, including the initiation, elongation, maturation and ultimate degradation of the membrane and its contents within the lysosomes. The class III PI3P kinase binds to Beclin 1 to promote the initiation of autophagy. Atg12-Atg5-Atg16L and LC3 conjugation systems contribute to the elongation of the isolation membrane and autophagosome closure. **B.** Autophagy-associated signaling pathways in cancer. Autophagy can be activated in response to multiple stresses during cancer progression, including nutrient deprivation, endoplasmic reticulum stress, hypoxia, glucose/energy depletion and other diverse stresses. The central signalling molecules in determining the levels of autophagy in cancer cells are AMPK-PI3K/Akt/mTOR pathways.

The term autophagy was a cellular ‘self-eating’ process by which intracytosolic components or organelles are sequestered into vacuolar compartments and subsequently delivered to the lysosome for bulk degradation and recycling [1, 2]. Currently, there are three major types of autophagy in eukaryotic cells, including macroautophagy, chaperone-mediated autophagy (CMA), and microautophagy [3]. Macroautophagy (hereafter referred to as autophagy in this review) is a multi-step process which regulates the turnover of cytosolic proteins and damaged or superfluous organelles. The autophagic process includes at least four distinct stages (i) initiation, which involves the formation of the phagophore through complex interaction of the autophagic protein Atg6 (Beclin 1) and class III phosphatidylinositol 3-kinase (PtdIns3K), along with intracytosolic components; (ii) elongation, where the phagophore is elongated and sequesters the cytoplasmic constituents by the microtubule-associated protein 1 light chain 3 (LC3; Atg8) and the Atg12-Atg5-Atg16L conjugation systems; (iii) maturation, resulting in encapsulation of cellular components which associates with complex; (iv) ultimate degradation of the engulfed components in the lysosomes (Figure 1A) [4, 5].

A series of signaling pathways have been implicated in autophagic process during cancer initiation and development. The central checkpoints in autophagy induction are the phosphatidylinositol 3-kinase (PI3K)/mammalian target of rapamycin (mTOR) and AMP activated protein kinase (AMPK) signaling pathways (Figure 1B) [6, 7]. Activated the class I PI3K generates phosphatidylinositol-3, 4, 5-triphosphate, which binds to the pleckstrin homology domain of Akt and PDK1 at the plasma membrane. As a result, Akt signaling pathway is activated. Once activated, Akt may stimulate multiple downstream targets, including the mTOR pathway which negatively regulates autophagy [8]. Conversely, AMPK, the primary energy-sensing kinase of the cell which is activated during hypoxia or energy depletion by increased ratios of AMP to ATP, can stimulate autophagy through mTOR in a TSC1/2 dependent pathway [9, 10]. In addition, endoplasmic reticulum (ER) stress may induce autophagy through the PERK-eukaryotic initiation factor 2α (eIF2α)-AEF4 and IRE1-JNK1 pathways [11, 12]. Hypoxia promotes the activation of autophagy through upregulation of BNIP3 [13]. Rag and Raf/MEK/ERK signaling pathways contribute to amino acid depletion induced autophagy [14]. DNA damage and energy depletion also respectively stimulate autophagy via p53 or LKB1 signal, the upstream target of mTOR.

Autophagy is involved in various cell aspects of biological processes, including cell survival, cell death, differentiation, senescence and metabolism [15]. Autophagy is constitutively active in the normal cells, which promotes the turnover of unfolded proteins and damaged organelles to maintain cellular homeostasis [16]. When the cells are subjected to stressful conditions including starvation, growth factors deprivation or high energetic requirements, autophagy can function as a survival signal to restore metabolic homeostasis and protect the cells from the damages from such conditions. However, persistent or excessive autophagy is also shown to promote cell death, termed autophagy-associated cell death or type II programmed cell death in contrast to type I programmed cell death or apoptosis [17, 18]. Recently, several studies have shown that autophagy also participates in the regulation of immune response. Autophagy may deliver cytoplasmic components to lysosomes, therefore, contribute to cytoplasmic immune recognition and response [19]. In addition, autophagy and autophagy-associated genes influence innate and adaptive immunity through regulating antigen processing and presentation [20]. Thus, autophagy is now widely recognized as an important regulator in immune recognition and responsiveness. However, how autophagy and autophagy-associated genes regulate antigen presentation and immune response is still less well acknowledged. Although the relationship between autophagy and cancer cell immune is quite complicated, and has not been well elucidated, understanding the novel role of autophagy may allow us to develop potential immunotherapeutic approaches against cancer.

## AUTOPHAGY AND CANCER IMMUNOLOGY

Recently, the role of autophagy has been expanded to immune systems, which in turn regulate innate and adaptive immune responses. Rapidly accumulating evidence has provided a link between stimulation of autophagy and cancer immune responses.

### Innate immunity-mediated autophagy and cancer immunology

**Figure 2:**
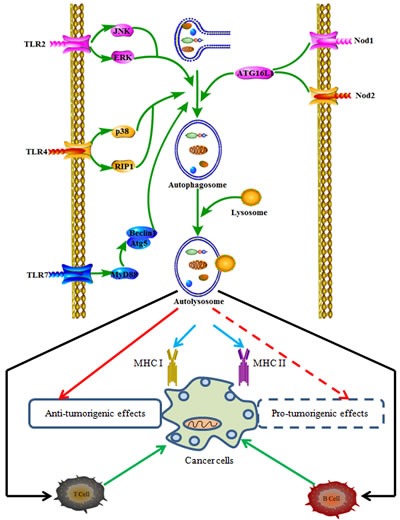
The relationship between autophagy-associated immune signals and cancer immune responses Activation of innate and adaptive receptors can regulate autophagy through different signaling pathways and act in the recruitment of autophagy proteins to phagosomal membrane, resulting in anti-tumorigenic effects or pro-tumorigenic effects.

The innate immune response of autophagy is mainly involved in the conjunction of pathogen-associated molecular patterns (PAMPs) and damage-associated molecular patterns (DAMPs) with pattern recognition receptors (PRR). PRR include Toll-like receptors (TLRs), nucleotide oligomerization domain (NOD)-like receptors (NLRs), and receptors for cytokines such as interferon (IFN), tumor necrosis factor (TNF)-α [21, 22]. Autophagy can be up-regulated by the activation of these innate immune receptors (Figure 2).

#### TLRs

TLRs are a series of innate receptors which are expressed in a wide variety of cancer cells and play a crucial role in several innate immune responses by regulating autophagy. TLR2 was reported to mediate phagocytosis and autophagy to enhance host innate immune response through the activation of c-jun N-terminal kinase (JNK) and extracellular signal-regulated kinase (ERK) signaling pathways [23, 24]. In response to lipopolysaccharide (LPS) or alpha-GalCer analog (CCL-34) treatment, TLR4 promoted macrophages through inducing expression of mitogen-activated protein kinases (ERK, JNK, p38 and RIP1) and some TLR4-downstream cytokines such as TNF-a, IL-6 and iNOS [25, 26]. TLR7 stimulated the autophagy in a myeloid differentiation factor 88 (MyD88)-dependent manner, resulting in eliminating intracellular microbes, even when the target pathogen was normally not associated with TLR7 signalling [27]. In addition to TLRs, TLR agonist pro-interleukin-1β (IL-1β) could be specifically sequestered into autophagosomes, whereas further activation of autophagy in antigen-presenting cells [28]. Taken together, TLRs and its agonists play an important role in the activation of autophagy. However, little is known about the potential interaction between TLR signaling and autophagy in cancer immunology. The DNA damage-regulated autophagy modulator DRAM1 functioned to mediate pathogen recognition by TLR-MYD88-NF-κB innate immune sensing pathway to activate selective autophagy [29]. Graphene oxide (GO), a nanomaterial with burgeoning bioapplications, may trigger autophagy to confer antitumor immune response through MyD88- and TNF receptor-associated factor 6 (TRAF6)-associated TLR-4/9 signaling pathways. Injection of GO alone into immunocompetent mice bearing the CT26 colon tumors not only suppressed the tumor progression but also enhanced autophagy and immune responses within the tumor bed [30]. Interestingly, there is a controversial report regarding the role of TLRs-associated autophagy in cancer development and immunology. The results from Zhan's laboratory demonstrated that the induction of autophagy contributed to TLR4- and TLR3-triggered progression of lung cancer cells by enhancing the production of chemokines and immunosuppressive factors via the promotion of TRAF6 ubiquitination [31].

#### NLRs

NLRs are a family of cytoplasmic molecules responsible for intracellular bacteria sensing and constitute an essential component of the host innate immune system. Nod1 and Nod2 are the first NLRs identified as microbial-associated molecular patterns (MAMP) detectors. Both Nod1 and Nod2 can directly stimulate autophagy by interacting at the plasma membrane with ATG16L1, an essential component for autophagosome formation [22]. Reports concerning the importance of NLR-mediated autophagy in neoplasia or possible cancer immune are very limited. Both Nod1 and Nod2 play a key role in realization of innate/adaptive immune responses and autophagy, and their gene polymorphisms may be associated with a variety of cancer types [32]. NOD1 is also an intracellular immune receptor that senses peptidoglycan from Gram-negative bacterial peptidoglycan (PG) and responds by inducing autophagy and activating NF-κB, leading to inflammation-mediated Helicobacter pylori (H. pylori) clearance which is considered as a important risk factor for gastric carcinogenesis [33].

#### Others

Recently, interferon regulatory factor 8 (IRF8) was reported to be a major regulator for autophagy maturation and innate immune responses through directly promoting autophagosome formation and lysosomal fusion [34]. IFN-γ was proved to trigger autophagy via p38 MAPK signaling pathway, which contributed to cell-autonomous innate immunity [35]. Importantly, IFN-γ can inhibit inflammation-associated gastric carcinogenesis by inducing epithelial cell autophagy and T-cell apoptosis [36]. Recent studies show that autophagy is also a crucial regulator of innate immunity cytokines such as interleukin (IL). Autophagy has a potentially pivotal role to regulate IL-23 secretion and innate T cell responses through effects on IL-1 secretion [37]. Autophagy also may modulate Borrelia burgdorferi-induced production of IL-1β and attenuation of IL-1β in immortalized cells is apparently a crucial step in viral immune evasion and initiation of malignancy [38, 39]. Melanoma differentiation associated gene-7(mda-7)/IL-24 is a unique member of the IL-10 gene family that stimulates antitumor immune response to promote cancer-targeted toxicity [40]. In addition, autophagic proteins LC3B and beclin 1 can regulate innate immune responses by inhibiting the release of mitochondrial DNA which is depended on the NALP3/NLRP3 inflammasome and mitochondrial reactive oxygen species (ROS) [41, 42]. Taken together, these studies have raised additional mechanisms that innate immune receptors-associated autophagy displays different regulation on cancer cells.

### Adaptive immunity-mediated autophagy and cancer immunology

#### Autophagy and antigen presentation

Cross-presentation is defined as a process by which the certain exogenous antigens are taken up, processed, and then presented to antigen-specific T cells by host professional antigen-presenting cells (APC), thus, cross-presentation is very critical for the induction of adaptive immunity [43]. Currently, autophagy has been identified to play a novel role in antigen sequestration and delivery to MHC class I molecules for cross-presentation [44]. Autophagy-mediated elimination was critical for MHC I-mediated cross-presentation and blocking autophagy resulted in a drastic increase in the number of intracellular bacteria and *Chlamydia*-positive DCs [45]. TNF-α was observed to induce autophagy to enable the processing and presentation of mitochondrial antigens at the cell surface by MHC class I molecules [46]. Several researches reported the relationship between MHC I-mediated autophagy and cancer immune response. Cross-presentation of tumor associated antigens by MHC class I molecules could be regulated by autophagy-mediated lysosomal proteolysis and proteasomal degradation [47]. The direct evidence from Li, et al. showed that autophagy was involved in the regulation of MHC-I antigen expression, through which autophagy could play different roles in tumor immunity: enhancing the cytolysis of CTL to melanoma cells at the early stage of melanoma, but impairing the cytolysis at the late stage [48].

Different from endogenous MHC class I peptides which are primarily generated by proteasomes in the cytosol, the endosomal/lysosomal system is responsible for the processing of MHC-II presentation. During MHC class II presentation, exogenous antigens are processed in endolysosomal compartments where they are degraded by lysosomal proteases and then fused with MHC class II loading compartment (MIIC), resulting in delivering cytosolic proteins to MHC class II molecules for immune surveillance [49]. Autophagy can deliver cytoplasmic material to lysosomes, therefore, contributes to cytoplasmic antigen presentation of MHC class II molecules [50]. Targeting of the Influenza Matrix Protein 1 (MP1) to autophagosomes via fusion to the autophagosome-associated protein Atg8/LC3 led to strongly enhanced MHC class II presentation to CD4^+^ T cell clones [51]. Similarly, autophagy may subject the cell to an enhanced immune surveillance by CD4^+^ T cells in response to stressful conditions [52, 53]. These studies establish clear evidence that autophagy facilitates MHC-II presentation of peptides from intracellular proteins in general way and indicate that autophagy might act as a potential mechanism for the cross-presentation of tumor antigen onto MHC molecules. However, how regulation of the autophagy pathways in cancer cells influences MHC molecules has not yet been thoroughly investigated.

#### Autophagy in the development and function of T cells and B cells

Beyond its role in antigen presentation, autophagy also shapes adaptive immunity through its contribution to the development and function of lymphocytes including T cells and B cells.

#### Autophagy in T Cells

T cells are particularly dependent on autophagy for their homeostasis and activation. Both CD4^+^ and CD8^+^ T cells are tightly regulated by positive and negative modulators of signaling pathways downstream of the T cell antigen receptor (TCR). Basal autophagy maintains organelle homeostasis in T cells and can be induced following TCR stimulation [54, 55]. Moreover, several autophagy associated proteins are involved in the activation of T cells. The deletion of Atg3, Atg5, Atg7, Beclin-1 and the class III phosphoinositide 3-kinase Vps34, the positive mediators of autophagy, caused impaired autophagy and defective T cell homeostasis by inhibiting T-cell survival through quality control of mitochondria [54, 56, 57]. Autophagy had different effects in the cell survival of HIV-infected T cells and “bystander” T cells, and it was altered differently by HIV infection. HIV-1 viral infectivity factor can interact with LC3B and inhibit autophagy [58]. However, HIV-1 Tat protein was also able to stimulate autophagy through increasing BAG3 levels in human glial cells [59]. Increasing evidence has suggests a link between the deregulation of autophagy and regulatory T cell-controlling anticancer immunity. Autophagy was induced in CD4^+^ T cells via JNK and Vps34 and played an important role for the growth factor-withdrawal cell death [60]. Autophagy also partly regulated the helper T lymphocytes (HTLs) antitumor responses against tumors expressing c-Met [61]. However, autophagy has not only pro-death but also pro-survival roles in T cells depending on cellular contexts and stimuli. The expression of autophagy-associated proteins in the peripheral site of oral squamous cell carcinoma (OSCC) significantly correlated with an increase in the infiltration of T cells and several unfavorable clinicopathological parameters, which indicated that autophagy may actively mobilize immune cells toward the cancer bed and contribute to malignant potential and an unfavorable prognosis [62]. In KRas^G12D^-driven lung cancer, Atg5-regulated autophagy bestowed cancer cells a significant survival advantage and accelerated tumor progression by increasing oncogenesis maps to regulatory T cells [63]. Hypericin-based photodynamic therapy (Hyp-PDT)-induced autophagy dampened key processes that underlie immunogenic cell death (ICD) and the elicitation of anticancer immune responses by suppression of immune effectors CD4^+^ and CD8^+^ T lymphocyte, thus facilitated cancer cells escape from immunosurveillance [64]. Taken together, these studies have raised a critical role for T cells-associated autophagy in cancer immunology.

#### Autophagy in B Cells

Limited evidence exists that autophagy plays a complex role in B cells development and survival. The activation of autophagy was found to be a mechanism for survival of autoreactive B cells [65]. Moreover, Autophagy gene Atg5 was required for B cell development, B-1a B cell maintenance and plasma cell development and function [66, 67]. Atg5 deletion failed to transition between pro- and pre-B-cell stages in the bone marrow. RelA appeared to be pivotal in both classical and alternative activation pathways in Epstein-Barr virus (EBV)-transformed B cells, which was partly due to the induction of autophagy [68]. Tumor-derived autophagosomes (termed “DRibbles”) could induce B cell activation and the current study suggested that macrophages significantly enhanced DRibbles-induced B cell immune function by TLR4 and MyD88 signaling pathway [69]. Taken together, the current data from multiple groups have shown that autophagy functions as an important role in the development of certain subsets of B cells and B cell memory [70].

## AUTOPHAGY-MEDIATING CELL IMMUNITY AND CANCER IMMUNOTHERAPY

Currently, immune-based therapies are attracting more and more attention in cancer treatment [71]. A variety of cancer immunotherapies, including vaccines and adoptive cell transfer, have been clinically applied [72]. Autophagy, a dynamic process of protein degradation, has been recently linked to cancer immunotherapy. Targeting autophagy-dependent cross-presentation and immune responses may provide us a promising therapeutic approach for cancer treatment, although the mechanisms underlying immunogenic potential of autophagy have not been well elucidated (Table 1).

**Table 1:** Therapeutic compounds and targets that modulate autophagy-dependent immune responses

Compound	Effect on autophagy	Immunogenic responses	Mechanism	Types of cancer	References
α-Al_2_O_3_	inducer	cross-presentation	unknown	Lung cancer	73
Monobenzone	inducer	T cells	unknown	Melanoma	74
p62-encoding DNA vaccine	inducer	unknown	unknown	Melanoma, Lung cancer and so on	75
Farletuzumab	inducer	FRa	unknown	Ovarian cancer	76
DRibble	inducer	DCs, cross-presentation	CLEC9A	Lung cancer, Melanoma	77
MTX	inducer	DCs, T cells, ATP	PI3K, Akt	Melanoma	83
BcG/cWs	inducer	TLR	JNK	Colon cancer	84
IFN1	inducer	innate immune	JAK1-sTAT1 and ReLA	CML	85
CQ	inhibitor	DCs	Endosomal pathways	Breast cancer	92
HCQ	inhibitor	B cells, CTL	unknown	Lymphoma, Melanoma	93,94

### Autophagy as a pro-death mechanism

Autophagy as a pro-death signal in the abnormal tumor microenvironment is an important mechanism that suppresses tumor progression and enhances anti-tumor immunity and response to therapies. Alpha-alumina (α-Al_2_O_3_) nanoparticles could induce efficient autophagy-dependent cross-presentation through delivering antigens to autophagosomes in DCs, which contributed to tumor regression [73]. Monobenzone augmented the processing and shedding of melanocyte-differentiation antigens by triggering melanosome autophagy, which induced cytotoxic human melanoma-reactive T cells and resulted in tumor suppression [74]. Autophagy protein p62-encoding DNA vaccine was found to elicit antitumor and antimetastatic activity in five types of commonly used transplantable tumor models [75].

Basing on that certain cancer cells are highly dependent on folate metabolism, the functional mechanisms of MORAB-003 (farletuzumab), a humanized mAb against folate receptor alpha (FRa), was investigated. As a result, MORAB-003 induced sustained autophagy and suppressed cell proliferation in ovarian cancer models [76]. Recently, autophagosome-enriched vaccine named DRibbles, was demonstrated to eradicate 3LL Lewis lung tumors and significantly delay the growth of B16F10 melanoma, which is partly due to CLEC9A, a newly described C-type lectin receptor expressed by a subset of conventional DCs [77].

Conventional chemotherapies are particularly efficient when they elicit immunogenic cell death (ICD) which is accompanied by stereotyped molecular changes, including the pre-apoptotic exposure of calreticulin (CRT) on the cell surface, the lysosomal secretion of adenosine triphosphate (ATP) during the blebbing phase of apoptosis, and the post-apoptotic release of high mobility group box 1 (HMGB1) from dying cells [78, 79]. Autophagy upregulated the mannose-6-phosphate receptor (MPR) on the tumor cell surface and caused ICD via T cells activation [80]. Newcastle disease virus (NDV) infection was shown to prime adaptive antitumor immunity in orthotopic glioma through the induction of ICD as well as autophagy [81]. Autophagy may attract DCs and T lymphocytes into the tumor bed and promote the immunogenic release of ATP from dying cells, and autophagy deficits abolish the capacity of cancer cells to elicit an immune response [82]. Systemic chemotherapy with the anthracycline Mitoxantrone (MTX) reduced the growth of autophagy-competent melanomas but not autophagy-deficient melanomas and this growth-inhibitory effect could be abolished by the combined depletion of CD4^+^ or CD8^+^ T lymphocytes, indicating that autophagy-dependent anticancer immune response determined the efficacy of MTX in melanoma [83]. BovisBacillus calmette-Guerin (BcG/cWs) is an effective antitumor immunotherapy agent and treatment with BcG/cWs plus ionizing radiation (IR) resulted in the induction of autophagic cell death in colon cancer cells through JNK and TLR pathways [84].

Many cytokines are also closely related to the activation of autophagy and cancer immune. IFN-γ not only mediated responses to bacterial infection and autoimmune disease but also functioned as an important tumor suppressor to inhibit gastric carcinogenesis by inducing epithelial cell autophagy and T-cell apoptosis [36]. IFN1 has been extensively studied as a treatment for patients with chronic myeloid leukemia (CML), however, the mechanism of anticancer activity of IFN1 is not well understood. The results from Zhu, et al. showed that autophagy regulated IFN1-mediated cell death, and activation of JAK1-sTAT1 and ReLA signaling were required for this process [85]. These findings reveal immunogenic effect of autophagy and witnesses that autophagy may be evaluated as a novel target for cancer immunotherapies.

### Autophagy as a pro-survival mechanism

Besides its potential to induce cell death, a pro-survival role is indicated in the process of autophagy following anticancer treatments.

On the one hand, the induction of autophagy may impair the anticancer immune responses and avail tumor cells to escape immune surveillance, resulting in tumor growth and cancer progression. The inhibition of Atg7 prevented intestinal tumor initiation through enhancing anticancer immune responses of CD8^+^ T cells [86]. The treatment of autophagy inducer rapamycin can promote metastasis of 4T1 cells in a tumor resection mouse model and this effect may be reversed by treatment with a DC-based cancer vaccine [87]. Epithelial-to-mesenchymal transition (EMT) may promote cancer cell invasion and metastasis, but its impact on immune surveillance has not been explored. Recently, it was shown that EMT transition exhibited attenuation in cytotoxic T lymphocytes (CTL)-mediated tumor cell lysis along with the induction of autophagy, indicating EMT and autophagy function as conceptual realms for immunotherapeutic strategies to block immune escape [88].

On the other hand, autophagy also provides intrinsic resistance against anticancer therapy by reducing anticancer immune effector mechanisms. Autophagy plays a protective role against sepsis-induced T lymphocyte apoptosis and immunosuppression, thus, down-regulation of autophagy in T lymphocytes may result in enhanced apoptosis and decreased cell survival [89]. The activation of autophagy in hypoxic melanoma cells was proved to selectively degrade gap-junctional channel activity of connexin 43 (Cx43), leading to the destabilization of the immune synapse and the suppression of NK cell-mediated tumor cell killing [90]. The inhibition of autophagy augmented cytotoxicity in combination with several anticancer approaches and inhibited cancer progression in preclinical models. Administration of high dose interleukin 2 (HDIL-2) has durable antitumor effects for the patients with melanoma and renal cell carcinoma. However, the side effects restricted its clinical application. Recent study indicated that inhibiting autophagy by chloroquine (CQ) enhanced the efficacy of HDIL-2 immunotherapy and decreased toxicity for cancer patients [91]. CQ also blocked radiation-induced autophagy and drove breast cancer cells into a more rapid apoptotic and more immunogenic form of cell death by Dcs, although T-cell stimulation was unaffected [92]. Moreover, anti-CD20 nanoparticles carrying Hydroxychloroquine (HCQ) and Chlorambucil increased tumor cell killing in comparison to free cytotoxic agents or Rituximab [93]. Hypoxia-induced resistance of lung tumor to cytolytic T lymphocyte (CTL)-mediated lysis was demonstrated to be associated with autophagy induction, and inhibition of autophagy by HCQ dramatically reduced tumor growth in B16-F10 tumor-bearing mice and restored the susceptibility of hypoxic tumor cell to CTL-mediated lysis [94]. In line with these observations, hypoxia-induced autophagy impaired cancer cell susceptibility to Natural killer (NK)-mediated killing and the activation of autophagy in hypoxic cells was involved in selective degradation of the pro-apoptotic NK-derived serine protease granzyme B. Inhibition of autophagy by targeting Beclin 1 restored granzyme B levels in hypoxic cells and induced tumor regression by facilitating NK-mediated tumor cell killing [95, 96].

These studies provide us a promising therapeutic strategy to suppress the development of cancer cells and enhance the effects of anticancer approaches to overcome the antitumor immune resistance.

## CONCLUSIONS AND PERSPECTIVES

Autophagy as a catabolic process has been implicated in multiple aspects of biological processes, including apoptosis, cell survival, senescence, metabolism and differentiation. Recently, several studies have shown that autophagy also participates in homeostatic regulation of immune responses. Advances over the past few years have expanded the scope of autophagy's role in immunity from its original incarnation as a catabolic mechanism to a full-range immunological process that participates in innate and adaptive immunity.

Currently, preclinical and clinical studies have identified the possible application strategy of autophagy as an immunotherapy. Autophagosome-enriched vaccines have been found to induced cytotoxic immune cells and elicit antitumor/antimetastatic activity [75, 77]. Targeting autophagy by nanoparticles or cytokines could serve as new therapies in the development of anticancer immunotherapy [73, 85]. In addition, optimal combination of autophagy-based inducer or inhibitor with various therapeutic strategies including chemotherapy, gene therapy, and other types of cellular therapies may represent an important approach by eliciting immunogenic cell death in current cancer therapeutics [73, 85].

Although some results from certain studies are encouraging, several fundamental questions about immunogenic regulation and autophagy remain to be answered. Firstly, the question whether we should try to enhance or inhibit autophagy in cancer immunotherapy is not straightforward since it might vary according to cell type and the cellular context. When and how autophagy can be pro-survival and pro-death should be carefully interpreted in the future. The second critical challenge is determining the roles of autophagy-mediated immune response and possible mechanisms. Although a large number of evidence supports that immunogenic cells are dependent on autophagy for their homeostasis and activation, biological functions and precise molecular mechanism that is involved in the process remain unclear. Thirdly, the treatment of CQ or HCQ can inhibit autophagy-related survival function and exert their anticancer action in combination with several immunotherapeutic strategies. However, the anticancer ability of CQ and its derivative might not only be due to inhibit the final degradative step of autophagy. CQ or HCQ may also affect other pathways such as lysosomal membrane permeabilization [97, 98], which should be considered in the ongoing studies where CQ or HCQ are used as an autophagy inhibitor. A more detailed understanding the novel immunogenic function of autophagy will be critical in the future.
